# Extensive diversity of *Symbiochlorum*‐related algae from environmental sequences and culture strains supports the description of the new family Symbiochloraceae (Ulvophyceae, Chlorophyta)

**DOI:** 10.1111/jpy.70046

**Published:** 2025-06-17

**Authors:** Heroen Verbruggen, Sanqiang Gong, Kefu Yu, Kshitij Tandon, Francesco Ricci, Jiayuan Liang

**Affiliations:** ^1^ CIBIO, Centro de Investigação Em Biodiversidade e Recursos Genéticos, InBIO Laboratório Associado, Campus de Vairão Universidade do Porto Vairão Portugal; ^2^ Melbourne Integrative Genomics, School of BioSciences University of Melbourne Parkville Australia; ^3^ Guangxi Laboratory on the Study of Coral Reefs in the South China Sea, Coral Reef Research Center of China, School of Marine Sciences Guangxi University Nanning China; ^4^ Department of Microbiology and Immunology, Peter Doherty Institute for Infection and Immunity University of Melbourne Parkville Australia; ^5^ Monash University, Department of Microbiology Biomedicine Discovery Institute Clayton Victoria Australia

**Keywords:** 18S nuclear ribosomal DNA diversity, Ignatiales, Symbiochloraceae, *Symbiochlorum*, Ulvophyceae

## Abstract

The genus *Symbiochlorum*, initially described from a single strain isolated from a coral in the South China Sea, was shown to be a sister lineage of *Ignatius* within the green algal order Ignatiales. Its significant phylogenetic divergence from *Ignatius* raises the possibility of its classification as a new family. To further investigate this hypothesis, we conducted a more elaborate analysis of sequence diversity within the *Symbiochlorum* clade. We aligned the 18S nuclear ribosomal DNA gene sequences of newly isolated *Symbiochlorum* culture strains from coral in the South China Sea and environmental sequences from the Great Barrier Reef. Strains isolated from *Porites lutea* coral colonies exhibited morphological similarities to typical *S. hainanense* (CCTCC M2018096). Analysis of the 18S nuclear ribosomal DNA gene revealed substantial diversity in both the V4 and V9 regions of the gene, with sequences clustering into two distinct lineages. Lineage 1 (L1), represented solely by environmental sequences from Great Barrier Reef sediment samples, displayed high levels of sequence divergence (2.2%–5.8%), suggesting it consists of multiple species. Lineage 2 (L2) included coral‐derived strains and environmental sequences from the South China Sea and the Great Barrier Reef, as well as an ascidian‐associated strain from Palau. The significant divergence between L1 and L2 (3.1%–9.1%) suggests they represent different genera. Based on these results, we propose the recognition of the new family Symbiochloraceae within the Ignatiales order.

**Abbreviations**
ASV: amplicon sequence variant
ITS: internal transcribed spacer
nrDNA: nuclear ribosomal DNA
PCR: polymerase chain reaction
SEM: scanning electron microscopy
TEM: transmission electron microscopy

## INTRODUCTION

The Ulvophyceae, a class of green algae (Chlorophyta), is commonly observed as seaweeds in marine environments (Cocquyt et al., 2010). The emergence of molecular methods has significantly advanced the reconstruction of the Ulvophyceae phylogeny (Hou et al., 2022; Jackson et al., 2018; Leliaert et al., 2012). Initial phylogenetic analyses using the 18S nuclear ribosomal DNA (nrDNA) gene suggested the Ulvophyceae form a monophyletic group, albeit with limited statistical support (Škaloud et al., 2013; Watanabe & Nakayama, 2007). A 10‐gene phylogeny based on nuclear and chloroplast genes also supported a monophyletic Ulvophyceae (Cocquyt et al., 2010), but subsequent studies based on whole chloroplast genomes have challenged the monophyly of the Ulvophyceae, indicating that several Ulvophycean lineages are closely related to other core Chlorophytan groups (Fang et al., 2017; Fučíková et al., 2014; Jackson et al., 2018; Sun et al., 2016).

Phylotranscriptomic analyses have partially corroborated these findings, suggesting a non‐monophyletic Ulvophyceae with the Bryopsidales emerging as sister to the Chlorophyceae, or indicating an unresolved complex relationship among the Chlorophyceae, Bryopsidales, and the rest of the Ulvophyceae (Del Cortona et al., 2020; Gulbrandsen et al., 2021). A recent study featuring nuclear genomic data, expanded taxon sampling, and advanced evolutionary models has supported three primary clades: the UUOI clade (including Ignatiales, Oltmannsiellopsidales, Ulotrichales, and Ulvales), the DS‐S clade (comprising Dasycladales and Scotinosphaerales), and the TC clade (encompassing Trentepohliales, Cladophorales, and Blastophysa; Hou et al., 2022).

Within the UUOI clade, the Ignatiales, often referred to simply as the *Ignatius* clade, is comprised of relatively overlooked and inconspicuous taxa that are primarily restricted to terrestrial habitats, including the genera *Ignatius* and *Pseudocharacium* (Cocquyt et al., 2010). The marine, coral‐associated genus *Symbiochlorum* was identified as a sister group to *Ignatius* (Gong et al., 2018). The divergence values for all nucleotide sequences studied so far (18S nrDNA gene, ITS rDNA region, *rbc*L gene) between *Symbiochlorum* and *Ignatius* have exceeded 11%, revealing a potentially ancient split between *Symbiochlorum hainanense* and *Ignatius*. In 2018, the *Ignatius* clade was formally described as a new family, the Ignatiaceae, within the newly established order Ignatiales (Škaloud et al., 2018). Although the significant phylogenetic and sequence differences suggested that *Symbiochlorum* could be classified as a new family, further comparative studies with a broader range of species were necessary to validate this idea.

In the present study, we aimed to expand our understanding of sequence diversity within the *Symbiochlorum* clade and assess its higher level classification. To this end, we constructed alignments of the 18S nrDNA gene, incorporating data from newly isolated strains sourced from various coral reef regions in the South China Sea, as well as environmental sequences from the Great Barrier Reef.

## MATERIALS AND METHODS

### Isolation, cultivation, and morphological examination of *Symbiochlorum*

Specimens of *Porites lutea* were collected from coral reefs in Daya Bay (22.83° N, 114.62° E) and the Nansha Islands (9.34° N, 115.12° E), in the South China Sea from 2018 to 2022. Healthy coral tissue samples, ~1 × 1 cm in size, were excised using a hammer and chisel and rinsed three times with filtered seawater. The coral tissue was homogenized using a pestle, and the homogenate was transferred into artificial seawater‐based f/2 medium (Slocombe et al., 2015) within a 250‐mL flask. The medium was supplemented with a KAS antibiotic cocktail consisting of 50 μg · mL^−1^ kanamycin, 100 μg · mL^−1^ ampicillin, and 50 μg · mL^−1^ streptomycin. Flasks were incubated at 27°C and illuminated with 30 μmol photons · m^−2^ · s^−1^ under a 12:12 h light:dark cycle. Subsequently, 200 μL of the f/2 liquid medium with algal cells released from coral tissue homogenate was plated onto solid f/2 medium (enriched with the KAS antibiotic cocktail) and incubated at 32°C with a light intensity of 30 μmol photons · m^−2^ · s^−1^ and a 12:12 h light:dark cycle. Colonies of algae that developed on the solid medium were selectively transferred into 10 mL of liquid f/2 medium in 50‐mL flasks. This process was iterated to isolate axenic algal strains. Cultures in the log growth phase were examined using light microscopy, scanning electron microscopy (SEM), and transmission electron microscopy (TEM; Gong et al., 2018).

### Sequencing of the 18S nrDNA gene of *Symbiochlorum* isolates

Total DNA was extracted from the *Symbiochlorum* isolates using the Qiagen DNeasy Plant Mini Kit (Qiagen, Hilden, Germany) according to the manufacturer's protocol. Amplification of the 18S nrDNA gene was performed with a set of universal primers (An et al., 1999). The polymerase chain reaction (PCR) amplifications were conducted according to the guidelines provided for the Pfu PCR master mix (Tiangen, Beijing, China) as described by Gong et al. (2018). Amplification products were purified using a PCR cleanup kit (Tiangen) and sequenced bidirectionally with the PCR primers via Sanger sequencing (Genewiz, China). The 18S nrDNA gene sequences of *Symbiochlorum* isolates were submitted to GenBank (PV460248‐53).

### Search for *Symbiochlorum* environmental sequences

Environmental sequences were obtained from two sources. First, we obtained two contigs from the assembly of a recently conducted RNAseq study of *Porites lutea* samples from Heron Island (Tandon et al., 2025). These contigs were generated by assembling RNA reads and filtered for host RNA, using Trinity v2.15.1 (Grabherr et al., 2011) with default settings. The resulting 18S nrDNA gene sequences showing similarity to *Symbiochlorum* were extracted and are provided in the Zenodo repository associated with this publication (https://doi.org/10.5281/zenodo.14194935).

Second, we obtained previously published 18S nrDNA sequences from diverse metabarcoding initiatives carried out on the Great Barrier Reef. This work used either the V4 or the V9 region of the 18S nrDNA gene. For the V4 region, we downloaded 64 datasets of the Australian Microbiome Initiative (2018) from the European Nucleotide Archive (https://www.ebi.ac.uk/ena) and called amplicon sequence variants (ASVs) with dada2 v.1.28 (Callahan et al., 2016) running in R v.4.3.3, cutting off the reads where the 6‐base rolling mean of quality fell below Q30 and removing primer sequences with cutadapt v.4.6. The error learning, sample inference, read merger, chimera removal, and ASV calling followed the standard dada2 workflow (https://benjjneb.github.io/dada2/tutorial.html). For the V9 region, data analysis was conducted using the data from Ricci et al. (2022) and followed that paper's analysis workflow based on dada2 and QIIME2 (Bolyen et al., 2019). The resulting ASV sequences are available from the Zenodo repository (https://doi.org/10.5281/zenodo.14194935).

### Phylogenetic analyses

For phylogenetic analyses, we utilized a comprehensive dataset of long (>1200 nt) reference sequences of the 18S nrDNA gene from representatives of relevant Ulvophyceae orders, along with Chlorophyceae and Trebouxiophyceae as outgroups. Some introns and previously described inserts (Kooistra et al., 2002) were excised from the sequences prior to analysis. These long sequences were combined with either V4 or V9 18S rDNA amplicon sequences and aligned with MAFFT v.7.309 with default settings (Katoh & Standley, 2013). The two resulting alignments, with lengths (1864 positions for V4 and 1863 for V9) predominantly determined by the long reference sequences, were subjected to phylogenetic analysis in IQtree v.2.2.2.6 using the TIM3e + I + R3 model as determined with the built‐in ModelFinder function (Kalyaanamoorthy et al., 2017; Minh et al., 2020) and 200 standard bootstrap replicates to assess statistical confidence in the tree topology. The two 18S rDNA gene alignments used for the phylogenetic trees are included in the Zenodo repository (https://doi.org/10.5281/zenodo.14194935).

## RESULTS

**FIGURE 1:**
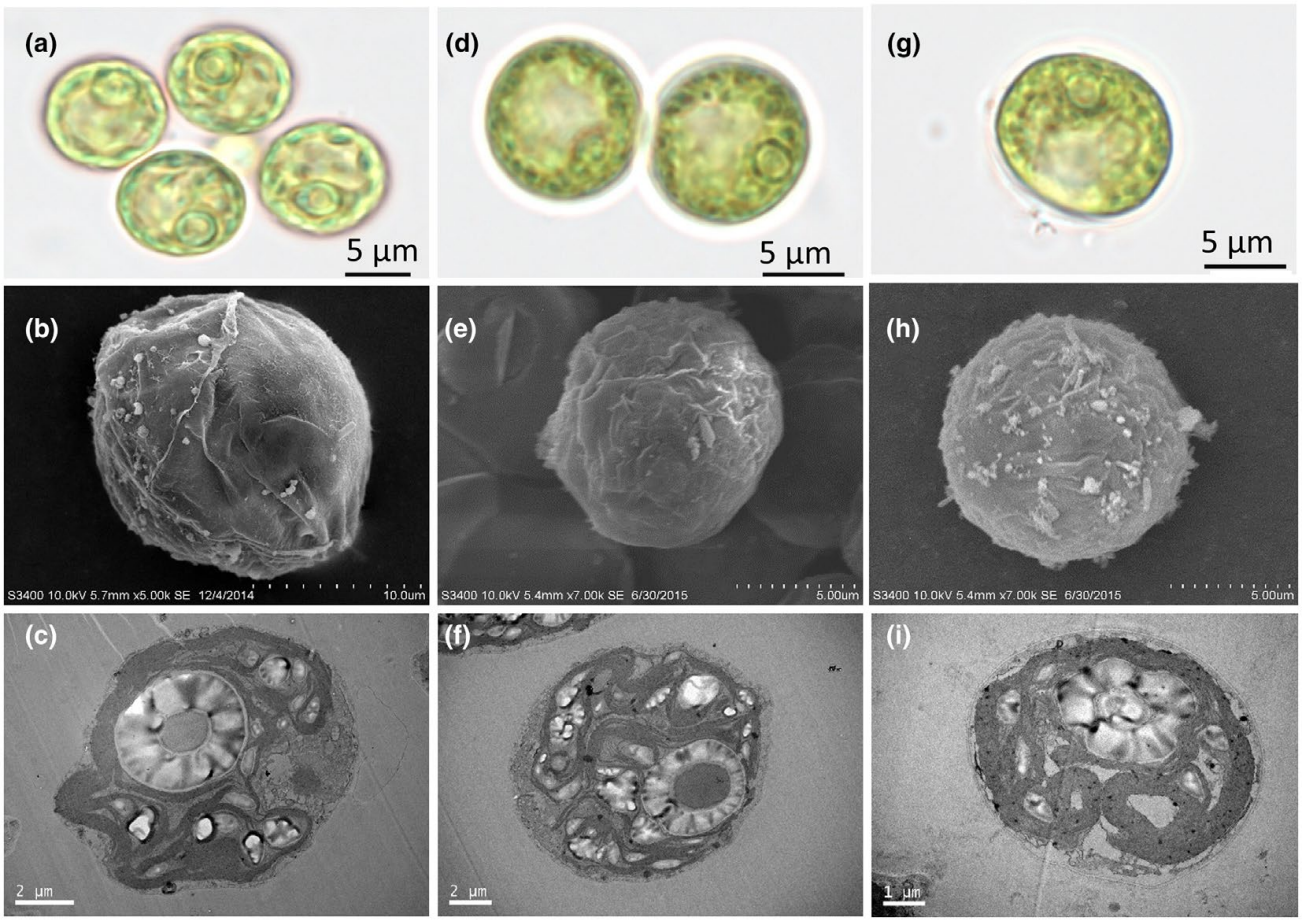
Light, scanning electron, and transmission electron microscopic presentations of vegetative cells of *Symbiochlorum hainanense*. (a–c) Type strain CCTCC M2018096 of *S. hainanense*. (d–f) Newly isolated strains from Daya Bay. (g–i) Newly isolated strains from Nansha islands. The top row shows light microscope images, the middle row single‐cell SEM images, and the bottom row TEM images.

The algal strains isolated from South China Sea *Porites lutea* specimens were morphologically similar to the type strain of *Symbiochlorum hainanense* (Figure 1). The vegetative cells of our *S. hainanense* strains were usually light green, with a visible cell wall, vacuole, nucleus, multiple chloroplasts with a pyrenoid, and were spherical and unicellular, 5–12 μm in diameter (Figure 1).

**FIGURE 2:**
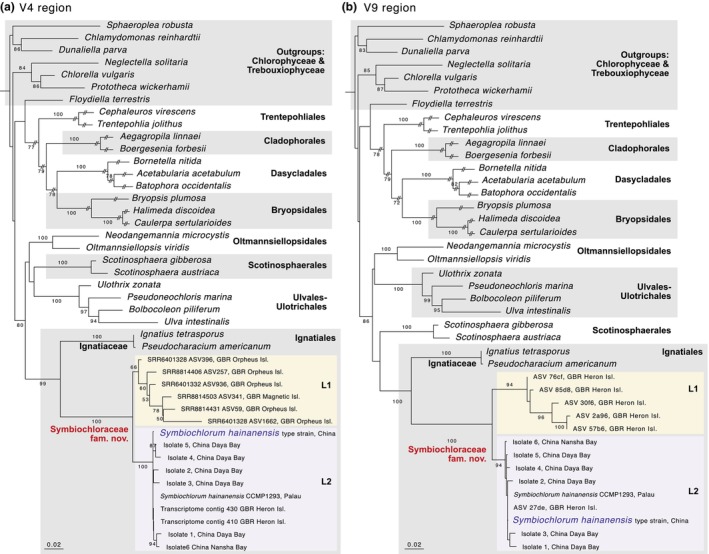
Phylogenetic analysis of *Symbiochlorum* 18S nrDNA sequences in the context of the main lineages of Ulvophyceae. (a) Analysis of the whole 18S nrDNA gene for samples where it was available, along with environmental sequences targeting the V4 region. (b) Analysis of the whole 18S rRNA gene for samples where it was available, along with environmental sequences targeting the V9 region. The scale is in estimated substitutions per site, but this scale does not apply to the lineage containing Trentepohliales, Bryopsidales, Cladophorales, and Dasycladales, which has elevated substitution rates and branches were shortened for compact visualization. The trees with original branch lengths are available in the Zenodo repository (https://doi.org/10.5281/zenodo.14194935).

The phylogenies of the V4 and V9 regions of the 18S nrDNA gene showed a substantial level of diversity in the *Symbiochlorum* clade, with two clearly defined lineages, indicated as L1 and L2 in Figure 2. Lineage 1 was only represented by environmental sequences and exclusively observed in sediment samples from the Great Barrier Reef, with the V4 sequences showing its presence in inshore locations (Orpheus and Magnetic Island) and the V9 sequences showing this lineage also occurs further offshore (Heron Island). Lineage 2 included a combination of strains isolated from corals, strain CCMP1293 isolated from an ascidian, and environmental sequences originating from Great Barrier Reef coral samples. Sequence variability was comparatively low in L2, while L1 showed more variability for both the V4 and V9 regions (Table 1, Figure 2).

**TABLE 1:** Divergences within and between main *Symbiochlorum* lineages based on the V4 and V9 regions of 18S rRNA.

	V4 (425 nt)	V9 (315 nt)
Within L1	2.2%–5.8%	0.3%–3.8%
Within L2	0%–2.5%	0%
Between L1 and L2	3.1%–9.1%	4.4%– 7.3%

*Note*: The values shown are uncorrected *p*‐values. Note that these values were obtained for alignments trimmed to just the amplicon region, so they do not account for variability outside of these regions that is included in the phylogenetic analysis shown in Figure 2.

## DISCUSSION

Our results based on environmental DNA sequences and isolated strains highlight a substantially higher diversity of *Symbiochlorum*‐related algae than what is currently appreciated. The two lineages (L1 and L2) identified in our phylogenetic analyses of 18S nrDNA gene included the previously known *S. hainanense* in L2 alongside the entirely unknown lineage L1.

*Symbiochlorum hainanense* was previously shown to be associated with coral hosts in Sanya Bay (China; Gong et al., 2018) and with an ascidian host in Palau (Darienko & Pröschold, 2024). Our new data showed that this alga is likely widespread and abundant across shallow‐water environments of the Pacific Ocean. Our new culture strains showed its presence in *Porites lutea* corals across several reefs in the South China Sea, and the environmental sequences clearly confirmed its presence in various Great Barrier Reef corals. The sequence variability within *Symbiochlorum* L2 was notably low, with differences ranging from 0% to 2.1%. Moreover, the morphological features of newly isolated *Symbiochlorum* strains from *P. lutea* were consistent with the type strain of *S. hainanense* (CCTCC M2018096). A revised description of *S. hainanense* (CCTCC M2018096) was recently published by Darienko & Pröschold, (2024), providing new insights into the morphology and phenotypic plasticity of *S. hainanense*. The morphological parallels and minimal sequence divergence between the cultivated strains and environmental sequences indicates that they belong to the same species, which inhabits a diverse range of habitats, including scleractinian and ascidian hosts. A recent report of *Symbiochlorum hainanense* from hydrocorals in the southwestern Atlantic Ocean further confirms the widespread nature, the wide host range and the limited 18S nrDNA diversity of this species (Andrade Aiube et al., 2024).

Lineage 1 (L1) exhibited a significantly higher level of genetic diversity than L2, with nucleotide divergences in both the V4 and V9 nrDNA regions ranging from 0.3% to 5.8%. To put this in perspective, the more thoroughly studied family Ulvaceae (Kawai et al., 2021) exhibited 1.1%–2.1% divergence between genera, based on sequences of *Ryuguphycus* (LC507144), *Ulvaria* (AY303590), *Umbraulva* (AB426225), and *Ulva* (AF189079). The substantially higher genetic divergences within the L1 suggest the presence of several species within this lineage.

We also argue that the significant genetic divergence between L1 and L2 (3.1%–9.1%) indicates that they should probably be considered different genera. Lineage 1 was exclusively detected in shallow‐water sediment samples, suggesting distinct habitat preferences for the two lineages.

The collective evidence from (1) the profound divergence between the *Symbiochlorum* lineage and the Ignatiaceae, (2) the existence of two diverging lineages (L1 and L2) within the *Symbiochlorum* clade that likely represent different genera, and (3) the notion that several species‐level lineages appear to exist in lineage L1, support the recognition of a new family to accommodate the species of the *Symbiochlorum* clade. Since L1 is presently only known from environmental sequencing data, we base the diagnosis of the family primarily on the known genus *Symbiochlorum*, although the genetic component of the diagnosis also includes L1. We also provide emended descriptions of the genus *Symbiochlorum* and species *S. hainanense* based on the latest observations.

**Symbiochloraceae fam. nov**. Verbruggen & S.Q.Gong.

Description: Unicellular green algae with chloroplasts forming a hollow sphere with small perforations and containing one pyrenoid. Fully developed cells contain several pyrenoids. Reproduction occurs through zoospores and aplanospores. In 18S nrDNA gene phylogenetic analyses, the Symbiochloraceae form a clade sister to the Ignatiaceae, with the sequences used for the phylogeny shown in Figure 2 serving as references.

***Symbiochlorum*** S.Q.Gong & Z.Yong Li emend.

*Description*: Unicellular algae consisting of cells ranging in shape from broadly ellipsoidal up to spherical when young. Chloroplasts numerous, parietal, forming a layer with small perforations, and containing one pyrenoid. The chloroplasts of fully developed cells become net‐like and contain several pyrenoids. The cells are uninucleate. The cell wall is relatively thin and becomes thicker with age. Reproduction occurs through zoospores and aplanospores. Zoospores are quadriflagellated with an anterior stigma. The chloroplasts of the zoospores are parietal and slightly perforated, with one pyrenoid. The zoospores are surrounded by thin cell walls and become spherical after moving.

***Symbiochlorum hainanense*** S.Q.Gong & Z.Yong Li emend.

*Description*: This alga appears widespread and abundant in Pacific shallow waters, supported by its presence in *Porites lutea* corals across South China Sea reefs and environmental sequences from Great Barrier Reef corals. Young cells are broadly ellipsoidal up to spherical and 5.4–8.8 × 4.0–7.7 μm in diameter. Initially, the chloroplasts form a hollow sphere with an opening with small perforations and contain one pyrenoid. After the cells reach ~7.0 μm in diameter, they start to form net‐like chloroplasts and possess two to three pyrenoids, but the hollow sphere can still be observed. The chloroplasts of the mature cells are net‐like with invaginations, where the pyrenoid is often located. Fully developed cells can contain several (from two up to seven) pyrenoids. Pyrenoids are well visible and are surrounded by large starch grains. The mature cells are spherical and 14.5 up to 27 μm in diameter or broadly ellipsoidal at 13.9–27.5 × 12.4–19.3 μm in size, and they are uninucleate. The cell wall is ~0.4–0.5 μm thick. The color of the chloroplasts is brownish. The largest cells are spherical, up to 26.0–27.0 μm in diameter. The cell wall becomes up to 1.3 μm thick. Reproduction occurs through zoospores and aplanospores. Zoosporangia can contain two to eight cells. Zoosporangia are spherical or broadly ellipsoidal and are 13.4–16.0 × 12.4–15.9 μm in size. Spores are released through the rupture of the sporangial cell wall. Zoospores are quadriflagellated and 5.9–6.1 × 3.5–3.7 μm in size. The flagella are 1.5–2.0 times longer than the body of zoospores. The stigma is anterior, and the cells are unnucleated. The chloroplasts are parietal, slightly perforated, and have one pyrenoid. The zoospores are surrounded by thin cell walls and become spherical after moving.

*Holotype*: Ultrathin sections deposited in Shanghai Jiao Tong University, Marine Biotechnology Laboratory (MBLZ 2017001).

*Culture strain*: The strain from which the holotype derives is accessioned in the China Center for Type Culture Collection (CCTCC M2018096).

*Nomenclature*: The species was initially described as *Symbiochlorum hainanensis* (Gong et al., 2018), but as the genus name is neutral, the correct epithet is *hainanense*. This is corrected here and is also how the species is listed in AlgaeBase.

## AUTHOR CONTRIBUTIONS

**Heroen Verbruggen:** Conceptualization (equal); data curation (equal); formal analysis (lead); funding acquisition (equal); investigation (equal); methodology (equal); project administration (equal); visualization (equal); writing – original draft (equal); writing – review and editing (equal). **Sanqiang Gong:** Conceptualization (equal); data curation (equal); formal analysis (supporting); funding acquisition (equal); investigation (lead); methodology (equal); project administration (equal); visualization (equal); writing – original draft (equal); writing – review and editing (equal). **Kefu Yu:** Investigation (supporting); methodology (supporting); writing – review and editing (supporting). **Kshitij Tandon:** Formal analysis (supporting); resources (supporting); writing – review and editing (supporting). **Francesco Ricci:** Formal analysis (supporting); resources (supporting); writing – review and editing (supporting). **Jiayuan Liang:** Investigation (supporting); resources (supporting); writing – review and editing (supporting).
